# Which climate change path are we following? Bad news from Scots pine

**DOI:** 10.1371/journal.pone.0189468

**Published:** 2017-12-18

**Authors:** Pierluigi Bombi, Ettore D’Andrea, Negar Rezaie, Mario Cammarano, Giorgio Matteucci

**Affiliations:** 1 Institute of Agro-environmental and Forest Biology, National Research Council of Italy (CNR-IBAF), Monterotondo, Italy; 2 LifeWatch-ITA, Mediterranean Thematic Center, National Research Council of Italy, Rome, Italy; 3 Institute for Agricultural and Forestry Systems in the Mediterranean, National Research Council of Italy (CNR-ISAFOM), Ercolano, Italy; Aristotle University of Thessaloniki, GREECE

## Abstract

Current expectations on future climate derive from coordinated experiments, which compile many climate models for sampling the entire uncertainty related to emission scenarios, initial conditions, and modelling process. Quantifying this uncertainty is important for taking decisions that are robust under a wide range of possible future conditions. Nevertheless, if uncertainty is too large, it can prevent from planning specific and effective measures. For this reason, reducing the spectrum of the possible scenarios to a small number of one or a few models that actually represent the climate pathway influencing natural ecosystems would substantially increase our planning capacity. Here we adopt a multidisciplinary approach based on the comparison of observed and expected spatial patterns of response to climate change in order to identify which specific models, among those included in the CMIP5, catch the real climate variation driving the response of natural ecosystems. We used dendrochronological analyses for determining the geographic pattern of recent growth trends for three European species of trees. At the same time, we modelled the climatic niche for the same species and forecasted the suitability variation expected across Europe under each different GCM. Finally, we estimated how well each GCM explains the real response of ecosystems, by comparing the expected variation with the observed growth trends. Doing this, we identified four climatic models that are coherent with the observed trends. These models are close to the highest range limit of the climatic variations expected by the ensemble of the CMIP5 models, suggesting that current predictions of climate change impacts on ecosystems could be underestimated.

## Introduction

Global climate is changing and it is influencing many aspects of human societies and natural ecosystems [1–3]. In order to plan effective adaptation strategies, global scenarios of climate conditions at medium and long term are needed. As a response, a large number of climate modelling centres around the world participated in the Coordinated Modelling Intercomparison Project Phase 5 (CMIP5), which represented the basis for the Fifth Assessment Report of the Intergovernmental Panel on Climate Change (IPCC-5^th^AR)[4]. Models used in the IPCC-5^th^AR are Global Climate Models (GCMs) based on four scenarios, denoted Representative Concentration Pathways (RCPs). They are identified by the total radiative forcing in year 2100 relative to 1750: 2.6 W/m^2^ for RCP2.6, 4.5 W/m^2^ for RCP4.5, 6.0 W/m^2^ for RCP6.0, and 8.5 W/m^2^ for RCP8.5. These four RCPs include one mitigation scenario (RCP2.6), two stabilization scenarios (RCP4.5 and RCP6.0), and one scenario with very high greenhouse gas emissions (RCP8.5). The goal of working with scenarios is to better understand uncertainties for taking decisions that are robust under a wide range of possible future conditions [5].

These models provide a wide range of predictions, which are priceless for estimating the potential alternatives on the ground [4], what human activities can produce, and what the inhabitants of the earth must face. Nevertheless, the range of available scenarios is so large that the uncertainty associated to comprehensive predictions may hamper the understanding of what is actually happening and the deriving decisions are robust but often ineffective. This is particularly true when decisions regard adaptation strategies for reducing the impacts of climate change on natural ecosystems. Therefore, it would be important to reduce such uncertainty, identifying which specific scenarios describe and predict the real climatic trend. This would allow to clarify with higher confidence the effect on nature and humankind and to plan more effective strategies.

Several studies evaluated the predictive performances of climate models and their ability to cope the current climatic trend. The IPCC-5^th^AR itself, in its 9^th^ chapter [6], reviewed the efforts to evaluate the performance of the CMIP5 models, both individually and collectively. Basically, the most direct and simple strategy to evaluate a model is to compare its predictions with measured values of the same quantities (e.g., global temperature, cumulative precipitation, solar radiation), as generally done by climatologic literature [7–9]. Furthermore, other property of CMIP5 climate models have been tested, such as the cross-scale properties of models [10], or uncertainty in responses to climate projections, such as carbon balance alterations [11]. Nevertheless, to the best of our knowledge, no research was focused on quantifying how well the available models explain the occurring variations in natural ecosystems and on identifying which specific scenario does it better.

There is ample evidence that modern climate change has a strong influence on plant and animal species world-wide [1,12,13]. The influence of changing climate on flora and fauna regard several aspects of species biology (e.g. phenology, physiology, interspecific interactions) [14–16]. In particular, effects on species geographic distributions were widely studied and documented [17–19]. This implies changes in marginal populations, which are exposed to less suitable conditions than those in central sites and are expected to have lower density, fitness, and genetic diversity [20–24]. In this perspective, the range shift of a given species in response to climate change could be observed at a certain time by measuring population dynamics in central and marginal sites.

This approach to range shift observation involves monitoring dynamic characteristics of the populations, such as population density, reproductive success, condition of the individuals, or other fitness-related traits whose variation can be interpreted as a response to climate change [25]. Positive responses indicate an improvement of local suitability associated with the range leading edge [26–28], neutral responses is a sign of no major variations in local suitability of core sites, and negative responses highlight the reduction of suitability in trailing edge sites [29,30]. On the basis of the spatial arrangement of these responses, it is possible to detect any variation of species range compatible with climate change. On the other hand, it is also possible to validate any prediction of climate change effects on species distribution by verifying whether the spatial pattern of observed responses is coherent with the expected variation in habitat suitability.

**Fig 1:**
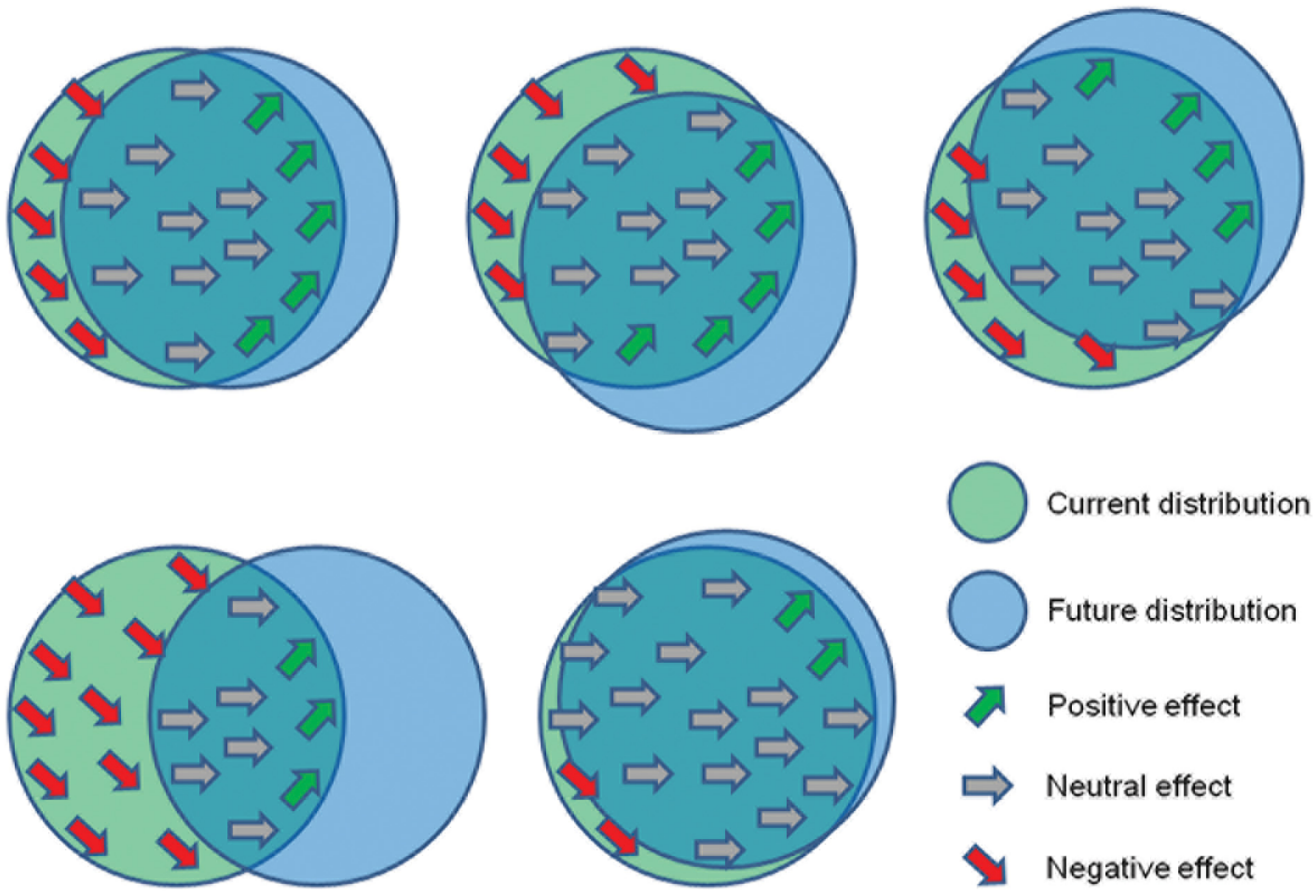
Schematic idea behind the experiment. Five hypothetical range shifts are shown with the relative expected effects on local populations. Let’s suppose only one of the five hypothesized range shifts is actually occurring. In the case an appropriate set of local data on population responses being available, it is possible to identify which specific range shift is actually occurring on the basis of the spatial pattern of positive, neutral, and negative responses.

Our hypothesis is that, if an appropriate set of local data on population responses is available, it is possible to identify which specific range shift is actually occurring, indicating which actual path of climate change the species is responding to. In other terms, we can validate the different GCMs on the basis of the spatial arrangement of positive, neutral, and negative responses in population dynamics (Fig 1), allowing to substantially reduce the uncertainty on future predictions of global climate. In order to verify our hypothesis and to provide foresights about future climatic trends, we adopted a multidisciplinary approach based on the comparison of observed and expected spatial patterns of response to climate change. More specifically, we utilized dendrochronological data to define growth variations for European trees in a set of sites and we compared this variations with those expected from an Ecological Niche Modelling approach for the same species. Doing this, we identified which specific models, among those included in the CMIP5, catch the real climate variation driving the response of natural ecosystems. In addition, we quantified the extent of the potential improvement in habitat suitability modelling when our criterion is adopted.

## Methods

### Experimental design

In order to identify which specific GCM explains the observed pattern of response by ecosystems, we utilized the recent growth variation for three species of European trees. For each species, we used dendrochronological data from several sites to define the observed spatial pattern of growth variation and an approach based on ecological niche modelling to define the spatial patterns of habitat suitability variation expected under each GCM. Then, we compared the observed pattern of growth variation with the expected patterns of suitability variation in the same sites, using a process based on null-models. The level of agreement between expected and observed patterns was assessed and the GCMs with the highest agreement were identified. These GCMs are those that better describe the specific changes which the trees are actually responding to. Therefore, these GCMs are those indicating the most probable pathways the ecosystems, and the humankind, are on.

### Observed ecosystem changes

**Fig 2:**
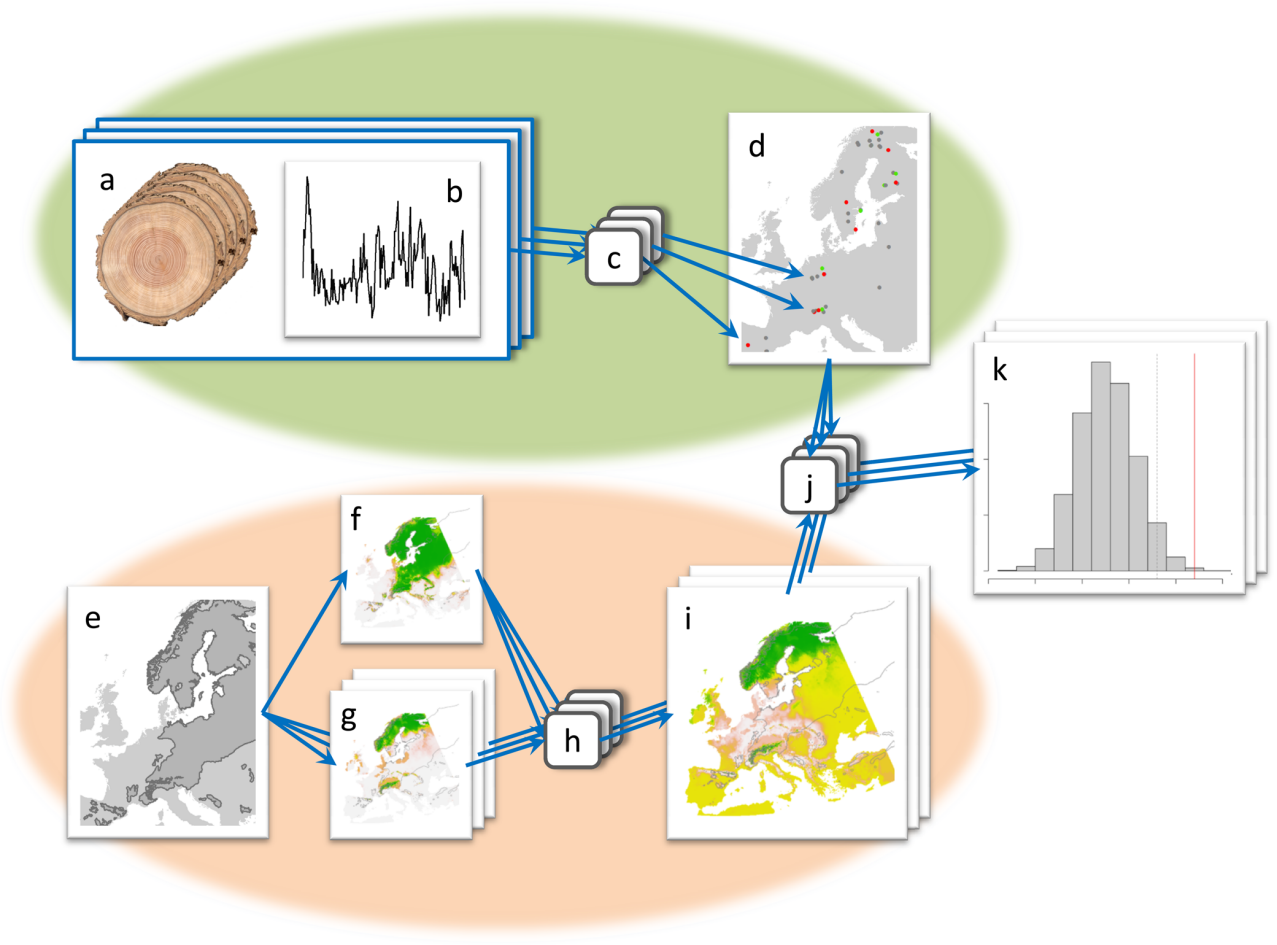
Workflow of our approach to model evaluation. The green ellipse highlights the dendrochronological process that from tree ring data (a) generates site-specific chronologies (b) and, through the comparison of recent and past growth (c), defines the pattern of observed responses (d). The pink ellipse highlights the niche modelling process that from the species distribution (e) provides: first the current climatic suitability (f) and one forecast of future suitability for each GCM (g); than, from the differences between future and current suitability (h), defines the GCM-specific patterns of expected responses (i). The comparisons between the observed and the expected patterns of responses (j) provide an evaluation of how well each GCM explains the current species response (k).

To define the observed pattern of response for each tree species, we adopted a dendrochronological approach. Site-specific series of average growth were created from measures of tree-ring widths and the recent growth was compared with that of the previous century. This allowed to define whether local populations are growing faster, slower, or as fast as in the past and to obtain a pattern of observed growth variations for each tree species. The sequence of favourable and unfavourable climate is faithfully recorded by the sequence of wide and narrow annual rings in large numbers of tree species [31]. This pattern of wide and narrow rings can be used as an indicator for monitoring environmental processes in most regions around the world [32]. First, measures of tree-ring widths (Fig 2a) were analyzed for creating site-specific series of average growth (Fig 2b). Second, for each site we compared the average growth of the most recent decade with that of the previous century (Fig 2c) through a null-model approach [33]. Third, we assigned positive observed trends to sites where the recent growth is significantly faster than before, negative trends to sites where the recent growth is slower than before, and neutral trends to sites where the recent growth is neither faster nor slower than before. Doing this, we obtained a spatial pattern of observed growth trends for each tree species (Fig 2d).

Tree ring data were obtained by The International Tree-Ring Data Bank (ITRDB) [34], which is the world largest public archive of tree ring data, managed by the Paleoclimatology Team at the National Centres for Environmental Information (NCEI) and the World Data Centre for Paleoclimatology at the National Oceanic and Atmospheric Administration (NOAA). The ITRDB contains tree ring data from samples collected in the field by using an increment borer to extract a wood core of 5mm in diameter along the radius of the tree. Generally, two cores from 20 trees of the same species were collected in each site [34]. From each core, series of tree-ring widths was measured with a variable resolution between 0.01 and 0.001 mm. All the series passed the standard dendrochronological methods of data cleaning and cross-dating [35]. Species-specific data for each sampling site are provided as a raw data file, containing all the measurements of the tree ring widths for each single series.

From the entire database, we selected data from European sites (west of 40.2° E) with series ending after 2000. Then, we selected the species with data from at least 20 sites. We visually inspected the geographic distribution of species sites and ranges and excluded two species because they did not respond to the exigencies of the experimental design. First, we eliminated *Fagus sylvatica* L. because the sites in the ITRDB were clustered in few areas of the species range. Second, we discarded *Larix decidua* Mill. because of the too small size of the distribution. As a consequence, we considered series of ring width from all the available sites for three species (i.e. Norway spruce, *Picea abies* (L.) H.Karst.: 40 sites; Scots pine, *Pinus sylvestris* L.: 57 sites; Sessile oak, *Quercus petraea* (Mattuschka) Liebl.: 26 sites; see S1–S3 Tables). These species are largely distributed in the study area and represent a significant part of the economic wood exchange in Europe.

The downloaded raw series of ring width data for each site were detrended and the results were averaged into one site-specific chronology. Detrending allows samples with large differences in growth rates to be combined, and can be used to remove age-related effects and potential disturbance signals [31] on growth trends. The detrending process involves fitting a curve to the ring-width series and dividing each ring-width value by the corresponding curve value to generate a series of growth indices. To do this, we used a modified negative exponential curve [31] (or a linear model when the suitable nonlinear model could not be used) in the R-based [36] package ‘dplR’ [37]. The obtained index values are unitless, with a nearly stable mean and variance, allowing indices from numerous trees to be averaged into a site chronology. We combined the indices to produce a standardized site chronology using Tukey’s Biweight Robust Mean [38].

Finally, for each species we defined the response to climate change (positive, negative, or neutral) in the considered sites as follows. We compared the growth indices during the last decade in the site chronology with those of the previous century. We adopted a null-model based approach for this comparison, contrasting the average growth index of the last 10 years with the averages of 30,000 random selections of 10 years from the previous 100 years. When the recent growth was lower/higher than 5-th/95-th percentile of the randomized previous growths, we rejected the null hypothesis (corresponding to a neutral response) that the recent growth was drawn at random from the distribution of the randomized previous growths [39,40] and assumed that a negative/positive response occurred. Using this criterion, we assigned one response to each site, obtaining a spatial pattern of observed responses to climate change.

### Expected ecosystem responses to climate change

To define the spatial pattern of expected responses, we adopted an approach based on ecological niche modelling. First, we defined the species-environment interactions [41] from the current species distribution (Fig 2e), by fitting a set of models within an ensemble approach [42]. Second, we projected the defined interactions into climate surfaces for present conditions (Fig 2f) and for the different scenarios of future climate (i.e. GCMs) (Fig 2g). Third, we assigned negative responses to sites where future climate suitability is lower than current, neutral responses to sites where future climate suitability is similar to current, and positive responses to sites where future climate suitability is higher than current (Fig 2h). Finally, by combining all sites, we obtained the spatial pattern of expected response under every tested scenarios of climate change (Fig 2i).

The current species distributions were derived from the EUFORGEN databank [43]. The European Forest Genetic Resources Program (EUFORGEN) is a collaborative program among European countries coordinated by Bioversity International in collaboration with the FAO, aimed to fulfill the pan-European forest policy [44]. EUFORGEN provide distribution maps for 34 species of European trees compiled by experts, based on existing bibliography and other information sources. Distribution maps are supplied as polygons for the main range, plus points for dispersed localities. We generated 5,000 random points across the study area (i.e. Europe, west of 40.2° E) and assumed as presence sites those falling into the species range, or close to the dispersed localities (< 2.5 arc-minutes), and as absence sites those falling outside. These presence/absence data were used for fitting the habitat suitability models on current climatic conditions.

We used climate surfaces from the WorldClim databank [45] for defining the species-specific climatic niche. The databank consists of climate data with global coverage for 19 climatic variables interpolated using measures for the period 1950–2000. In particular, we used climate surfaces with pixels of 2.5 arc-minutes of geographic degree, corresponding to a resolution of approximately 5 km [46]. Climate variables were assigned to the presence/absence points and selected for reducing multicollinearity by discarding those with Variance Inflation Factor (VIF) > 8 [47]. After this selection eight variables were retained for modelling (see S4 Table).

Ecological modelling is associated to a certain degree of uncertainty coming also from the model building procedure itself [48]. For incorporating the model-linked uncertainty into the outputs, we adopted an ensemble modelling approach [42]. Therefore, we used different methods for calibrating models of climate suitability on species distribution data. In particular, we fitted Generalized Linear Models (GLMs) [49], Generalized Additive Models (GAMs) [50], Generalized Boosting Models (GBMs) [51], and Classification Tree Analyses (CTAs) [52] in the R-based [36] package ‘biomod2’ [53]. Each independent model was projected into the current climate across the study area and three-fold cross-validated by calculating the true skill statistic (TSS) [54] and the area under the receiver operating characteristic curve (AUC) [55]. Finally, we calculated the TSS-weighted sum of the independent models for generating the specific consensus model of current habitat suitability.

In order to predict the variation of climate suitability under future respect to current conditions, we projected the fitted models into the available Global Climate Models (GCMs). We used GCMs elaborated by 19 research centres for the four RCPs for 2050 (average for 2041–2060) and downscaled at the same spatial resolution as the current models (i.e. 2.5 arc-min) using WorldClim as baseline climate [45]. Overall, we considered 59 different GCMs (see Table 1 and S5 Table for a complete list) and we used the same model parameterizations as in the current conditions for projecting climate suitability into the future and for creating the final consensuses model. As a result, we obtained one single spatial prediction of future climate suitability for each GCM.

**Table 1:** Agreement between observed responses by tree ring analyses and expected responses by habitat suitability models under different GCMs for *Pinus sylvestris*.

Climatic Model	RCP2.6		RCP4.5		RCP6.0		RCP8.5	
	*a*_*obs*_	*P{A*_*sim*_ *> a*_*obs*_*}*	*a*_*obs*_	*P{A*_*sim*_ *> a*_*obs*_*}*	*a*_*obs*_	*P{A*_*sim*_ *> a*_*obs*_*}*	*a*_*obs*_	*P{A*_*sim*_ *> a*_*obs*_*}*
**ac**			20	0.600			21	0.166
**bc**	14	0.804	17	0.860	23	**0.059**	22	0.303
**cc**	19	0.614	20	0.603	25	0.101	20	0.369
**ce**			16	0.815				
**cn**	21	0.365	26	0.073			27	**0.056**
**gd**	20	0.427	21	0.536				
**gf**	21	0.355	21	0.545				
**gs**	22	0.306	24	0.343	23	0.167	24	0.100
**hd**	22	0.660	19	0.549	24	0.196	23	0.120
**he**			21	0.362			21	0.362
**hg**			21	0.164			19	0.488
**in**			18	0.725			24	0.129
**ip**	21	0.535	19	0.668	21	0.201	25	0.111
**mc**	23	0.168	22	0.468	25	0.102	25	***0*.*009***
**mg**	21	0.479	23	0.169	21	0.366	20	0.716
**mi**	21	0.257	24	0.130	24	0.099	27	***0*.*001***
**mp**	17	0.892	18	0.317			17	0.779
**mr**	22	0.103	21	0.212	23	0.103	22	0.213
**no**	19	0.668	25	0.188	20	0.602	18	0.724

Each table row shows the measured agreement (*a*_*obs*_) and the result of the null-model test of agreement (*P{A*_*sim*_ *> a*_*obs*_*}*) for one model and four RCPs. The measured agreement (*a*_*obs*_) is the number of sites where the observed coincides with the expected responses. The result of the null-model test of agreement (*P{A*_*sim*_ *> a*_*obs*_*}*) is the probability of the null hypothesis that the measured agreement (*a*_*obs*_) was drawn at random from the distribution of the simulated agreement (*A*_*sim*_). The four models with the highest level of agreement between expected and observed responses are highlighted in bold. Values in italic indicate models with *P{A*_*sim*_ *> a*_*obs*_*}* < 0.01. See S5 Table for model details.

The expected pattern of response (positive, negative, or neutral) under each GCM was defined by generating a map of suitability variation as the difference between future and current model predictions. From the map of variation, we identified the areas where climatic suitability is expected to increase, reduce, or remain almost the same by selecting one positive and one negative threshold of variation. Since the frequency distribution of suitability variation had very high kurtosis and skewness, we selected two thresholds corresponding to the medians of positive and negative values. Finally, the expected responses were assigned to the same sites of the observed responses on the basis of their location. Doing this, we assigned negative expected responses to sites falling in areas with decreasing suitability, neutral expected responses to sites in areas with almost constant suitability, and positive expected responses to sites in areas with increasing suitability. As a result, we obtained one spatial pattern of expected responses for each of the considered GCMs.

### Agreement between observed and expected trends

For each species, the spatial pattern of observed responses was compared with that expected under each different GCM. To do this, we tested the level of agreement between observed and expected responses in the same sites by adopting a null-model approach [33,40,56]. First, we defined the measured agreement (*a*_*obs*_) as the number of sites where the observed and the expected responses are spatially matching (i.e. both positive, both neutral, or both negative) (Fig 2j). Second, we generated 30,000 random permutations of the expected responses and calculated the simulated agreement with the observed responses for each permutation (*A*_*sim*_). Third, we calculated the probability of the null hypothesis (*P{A*_*sim*_ *> a*_*obs*_*}*) that the measured agreement was drawn at random from the distribution of the simulated agreements (Gotelli, 2000) (Fig 2k). In other words, the null hypothesis is that the measured agreement is obtained by chance from a random geographic pattern of expected responses. This probability is a measure of the level to which the responses expected on the basis of a given GCM explains the actual responses observed in the sites. When the null hypothesis can be rejected (*P{A*_*sim*_ *> a*_*obs*_*} < 0*.*05*), the specific GCM used for modelling explains the real pattern of tree growth variation occurring in Europe.

### Potential improvement in habitat suitability modelling

Identifying which specific GCMs explain the real responses of ecosystems allows to estimate the potential error committed using all the available GCMs in habitat suitability modelling by including those that do not represent the real pressure on ecosystems. To do this, we compared the consensus models of future suitability deriving from the entire set of predictions, based on all the 59 GCMs, and from the restricted set of models that explain the observed ecosystem responses. Consensus models were calculated as the average of the individual predictions. We calculated the *t*-test between suitability values predicted by all the 59 models and by the selected models in each and every raster cell. This means that we performed 434885 individual *t*-tests, comparing all the pixels with mean suitability value > 30. In order to maintain this calculation within reasonable time limit, we adopted a parallel computation approach using a cluster of seven processors in the R-based package ‘parallel’ [36]. This allowed us to find pixels where predictions of the entire set of models are significantly different from those of the selected models.

## Results

### Observed responses

The dendrochronological analyses identify sites with positive, neutral, and negative responses for all the three species. The Norway spruce, *Picea abies*, has positive responses in 18 sites, neutral responses in 18 sites, and negative responses in four of the 40 sites of this species (S1 Table). The Scots pine, *Pinus sylvestris*, has positive responses in 10 sites, neutral responses in 37 sites, and negative responses in 10 of the 57 sites of this species (S2 Table). The Sessile oak, *Quercus petrea*, has positive responses in three sites, neutral responses in 18 sites, and negative responses in five of the 26 sites of this species (S3 Table). Overall, the percentage of non-stable sites (i.e. with positive or negative responses) is pretty high, being 55%, 35%, and 31% for the three species, respectively. The non-stable sites have more often positive than negative responses for *Picea abies* (χ^2^ = 8.91, p < 0.01) but positive responses are as frequent as negative for *Pinus sylvestris* and *Quercus petraea*.

### Expected responses

Ecological niche models under current climatic conditions obtain very high validation scores for all the three species. Individual models for *Picea abies* have TSS and AUC values in the range 0.77–0.86 (mean 0.82) and 0.94–0.98 (mean 0.96) respectively and the consensus model for the same species have TSS = 0.88 and AUC = 0.98. Individual models for *Pinus sylvestris* have TSS and AUC values in the range 0.77–0.83 (mean 0.80) and 0.93–0.96 (mean 0.95), respectively and the consensus model for the same species have TSS = 0.85 and AUC = 0.97. Individual models for *Quercus petraea* have TSS and AUC values in the range 0.77–0.83 (mean 0.81) and 0.93–0.96 (mean 0.95), respectively and the consensus model for the same species have TSS = 0.85 and AUC = 0.98. These values guarantee good fittings and are solid bases for robust predictions.

Future predictions of species climatic suitability vary largely with the different GCM considered. The number of sites with negative expected responses lays between 10 and 21 (mean 15.63) for *Picea abies*, between 15 and 37 (mean 23.10) for *Pinus sylvestris*, and between 0 and 9 (mean 1.41) for *Quercus petraea*. The number of sites with neutral expected responses lays between 17 and 29 (mean 23.97) for *Picea abies*, between 15 and 29 (mean 22.09) for *Pinus sylvestris*, and between 14 and 23 (mean 21.59) for *Quercus petraea*. The number of sites with positive expected responses lays between 0 and 8 (mean 0.41) for *Picea abies*, between 1 and 20 (mean 11.81) for *Pinus sylvestris*, and is 3 for *Quercus petraea*. The mean percentage of non-stable sites (i.e. with positive or negative expected responses) is 40%, 61%, and 17% for the three species respectively. These values represent the GCM-specific expected responses that are compared with the observed ones.

### Trend agreement

For Norway spruce, *Picea abies* and Sessile oak, *Quercus petraea* the expected patterns of responses for all the GCMs do not match the actual pattern of tree growth variation (*P{A*_*sim*_ *> a*_*obs*_*}* > 0.05 for all GCMs). On the contrary, predictions of habitat suitability variation from four GCMs explain the observed pattern of responses for Scots pine, *Pinus sylvestris* (Table 1). More specifically, for two GCMs the agreement between expected and observed patterns is highly significant (*P{A*_*sim*_ *> a*_*obs*_*}* < 0.01) and for other two GCMs the agreement is almost significant (*P{A*_*sim*_ *> a*_*obs*_*}* ≈ 0.05). All the other GCMs do not provide any explanation to the occurring pattern of responses in European populations of *Pinus sylvestris*.

**Fig 3:**
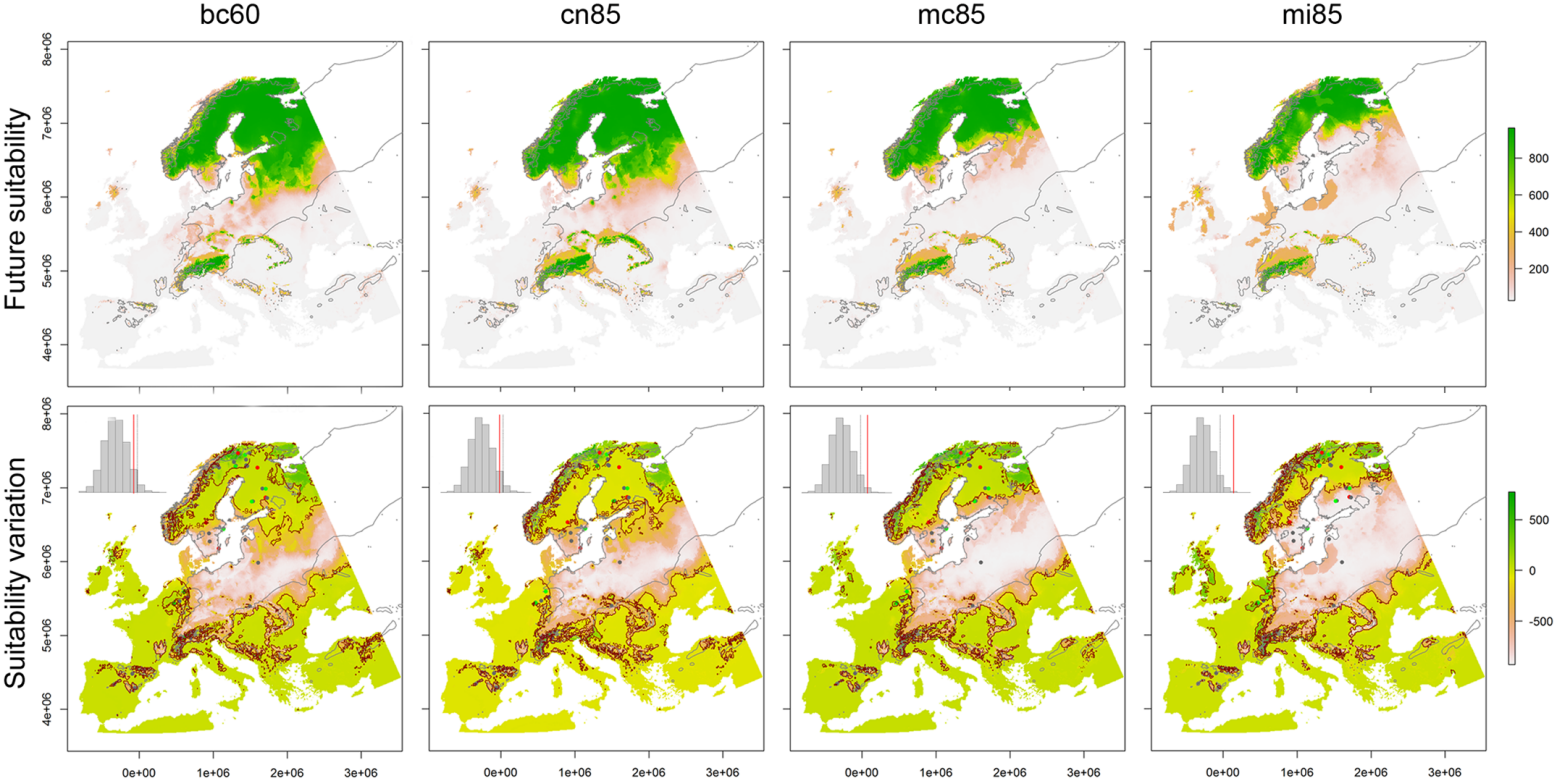
Models explaining the observed responses. Each column shows results for one of the four models with the highest level of agreement between expected and observed responses. In the upper row, the current range of *Pinus sylvestris* (thin grey line) and its future climate suitability are shown (grey-yellow-green scale; 0 < HS < 1000). In the lower row, the expected variation of climate suitability is shown (grey-yellow-green scale), together with the observed responses of *Pinus sylvestris* populations: green points represent populations with positive responses, grey points represent neutral responses, and red points represent negative responses. The small histograms within lower maps show the results of the null-model tests of agreement between expected and observed responses: when the vertical red line is on the left of the black line, *P{A*_*sim*_ *> a*_*obs*_*}* > 0.05, when the red line is on the right of the black one, *P{A*_*sim*_ *> a*_*obs*_*}* < 0.05. All the maps are Albers equal-area conic projections and coordinates are metric. See S5 Table for model details.

The four models explaining the spatial pattern of observed responses agree in predicting a substantial split of suitable areas for *Pinus sylvestris* in Europe (Fig 3). In particular, Fennoscandia and Alps are expected to remain climatically suitable even on the long run (i.e. 2050), while a strong reduction of suitability is expected to occur in central Europe with variable extent and intensity among the considered GCMs. This general pattern of geographic variation in climate suitability is common to all the best performing four models. Overall, the level of agreement increases with the strength and amplitude of the suitability reduction. Indeed, the best explanation of the occurring pattern corresponds to the largest contraction predicted for the main suitable areas and a small enlargement at the western range margin.

### Model identification

The GCM predicting the pattern of variation with the highest agreement with the occurring responses is MIROC-ESM-CHEM [57] ("mi" in Table 1) and the second best is MIROC5 [58] ("mc" in Table 1), both parameterized under the RCP8.5 (indicated in figures as "mi85" and "mc85" respectively). Both MIROC-ESM-CHEM and MIROC5 are global climate models developed by the University of Tokyo, the National Institute for Environmental Studies of Japan, and the Japan Agency for Marine-Earth Science and Technology for the phase 5 of the Coupled Model Intercomparison Project (CMIP5). The third and the fourth GCMs, which obtained almost significant agreements, are CNRM-CM5 [59] ("cn" in Table 1) at RCP8.5 ("cn85" in figures) and BCC-CSM1-1 [60] ("bc" in Table 1) at RCP6.0 ("bc60" in figures). These GCMs are models developed for the CMIP5 by the National Centre for Meteorological Research of France and the Beijing Climate Center, China Meteorological Administration, respectively. These four GCMs are those explaining the geographic pattern of growth variation in *Pinus sylvestris* populations across Europe with the best accuracy.

**Fig 4:**
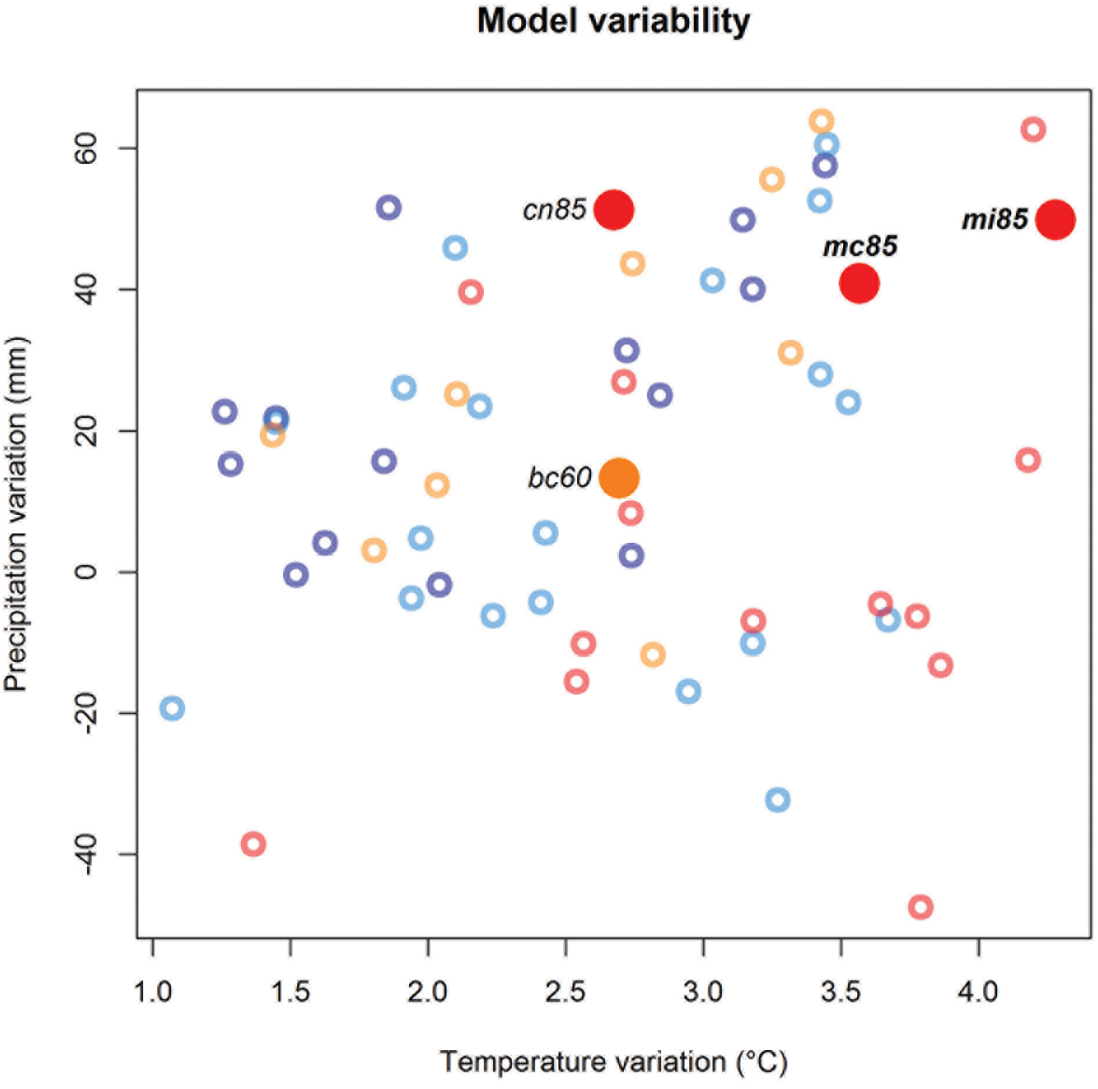
Variability of GCM predictions for 2050. Expected variation of annual mean temperature and cumulative annual precipitation in the study area by the different GCMs. The four large full points indicate models (acronyms reported) with the highest levels of agreement between expected and observed responses. Bold names highlight models with *P{A*_*sim*_ *> a*_*obs*_*}* < 0.01. Small empty points represent all the other models and the symbol colour indicate the Representative Concentration Pathways (RCP2.6: blue symbols, RCP4.5: azure symbols, RCP6.0: orange symbols, RCP8.5: red symbols).

Among the best four GCMs, the level of agreement with the observed pattern grows with the change intensity of climate scenario (Fig 4). Three of these models are based on the worst assumption of greenhouse gas concentration trajectory (i.e. RCP8.5). The fourth GCM is based on the RCP6.0 but, at the same time, it is the less significant among the best four (see Table 1). On the other hand, the very best explanation of the occurring pattern derives from the GCM predicting the highest temperature increase in the study area (i.e. mi85). Similarly, the second model to obtain significant agreement with the observed pattern predicts very high increases in air temperature and annual precipitation across Europe by 2050 (i.e. mc85). Our results indicate that these GCMs catch the climate variation which *Pinus sylvestris* is responding to better than the other CMIP5 models.

### Effects on habitat suitability modelling

**Fig 5:**
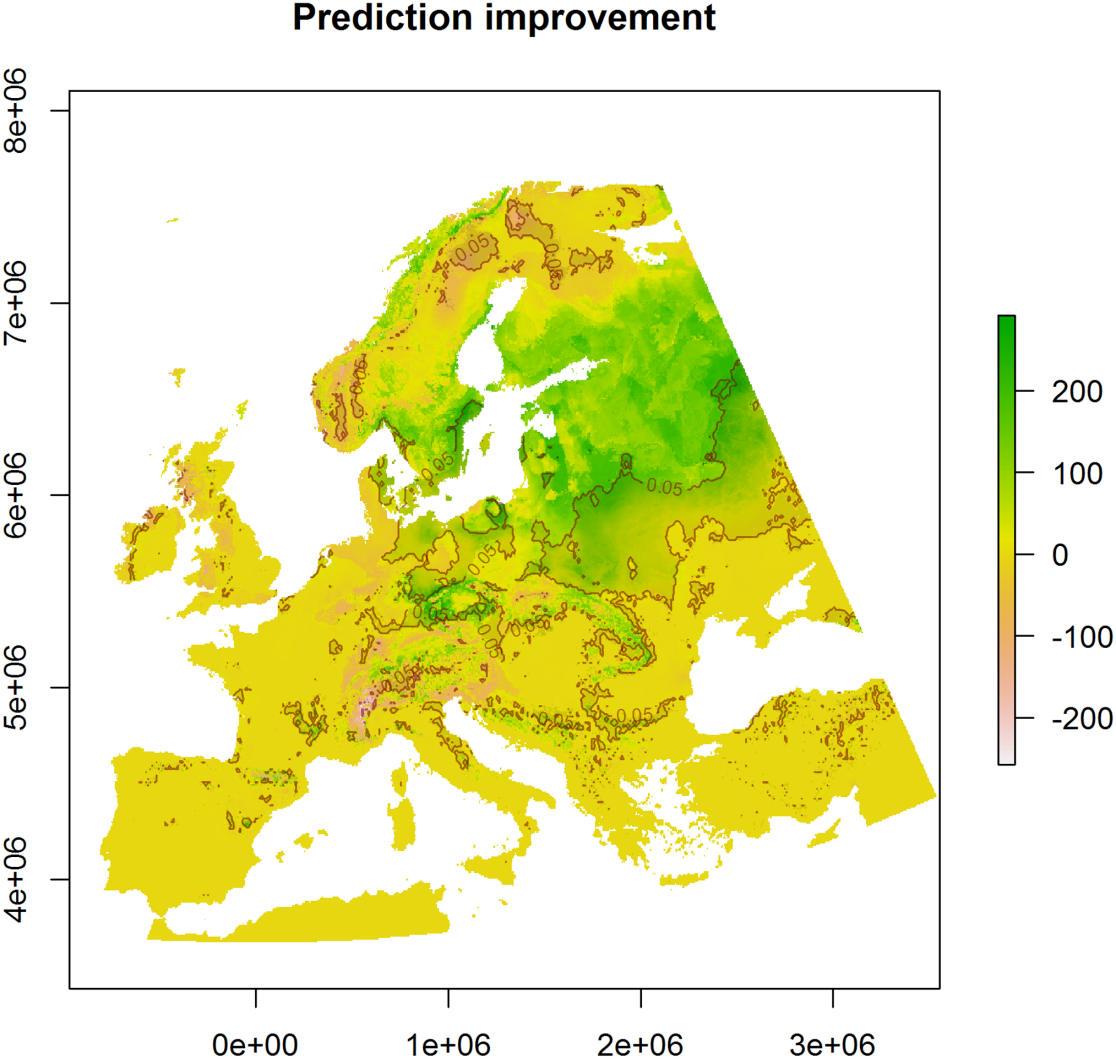
Potential improvement in future suitability predictions. Difference between habitat suitability under future climatic conditions as predicted by the entire set of models and by the selected four. The colour scale is the difference between the consensus model of the entire set of 59 models and the consensus of the selected four models. The darker area within the red line indicates where the difference is significant.

The difference between the consensus model of the entire set of 59 models and the consensus of the selected four models for *P*. *sylvestris* has a clear geographic pattern. This is particularly high in a large area of north-eastern Europe (see green areas in Fig 5) and particularly low on the Alps and in Scandinavia (see pink areas in Fig 5). As indicated by the pixel-based *t*-tests, the difference is significant in a large area of central and eastern Europe as well as in several smaller areas in Scandinavia, Alps, and Balkans (see darker areas in Fig 5). The future suitability for *P*. *sylvestris* is significantly higher when predicted by the entire set of models in an area of 1425251 km^2^ and it is higher when predicted by the four selected models in 202256 km^2^. These areas indicate the extent of the error potentially done by predicting future suitability including GCMs that do not represent the real pressure on ecosystems.

## Discussion

In this study we adopted a multidisciplinary approach, using methods and expertise from climatology, dendrochronology, and ecological niche modeling. We evaluated the performance of 59 GCMs with regard to the observed response of 123 forest sites across central Europe encompassing three species. Predictions from GCMs were translated into maps of expected response (predicted change in suitability) using a niche modelling approach, while the observed ecosystem responses were estimated using dendrochronological methods. Finally, we identified the GCMs that significantly explained the observed spatial pattern of ecosystem responses using a null model approach.

Our model-based expected responses suggest that the three species will have different shifts in the years to come, as indicated by the variable number of sites with positive, neutral, and negative expectations. However, the observed responses, derived from dendrochronological data, support the predictions of four models for *Pinus sylvestris* only. These four models could have several applications. For instance, they could be useful in adaptive forestry [61]. Indeed, the cultivation of *P*. *sylvestris* in central Europe, where climate suitability is expected to decrease, could become less efficient than in the past and questionable in the long run. Interestingly, the four significant matches between observed and expected pattern only occurs for *Pinus sylvestris* and, even more interestingly, all involves GCMs among those with the worst prediction in terms of global warming. In other words, we found the fingerprint of a particularly dangerous scenario of climate change in European forests.

All the other predictions are not in agreement with the evidences from measured tree growth. It means that these unsupported models did not catch the real processes occurring in the considered European ecosystems. Several potential factors might prevent the identification of tree responses to climate change. These factors can be linked to the nature itself of the response, to the quality of the available data, to the uncertainty associated with the process of dendrochronological analysis and ecological modelling, and to many other aspects. All these factors can be simultaneous and interacting, amplifying the error and further reducing the sensitivity of our approach. In addition, these factors can act in one or more phases of our approach to tree response detection blurring the observed pattern, undermining the predicted response, or attenuating the agreement. In all these cases, the probability of identifying significant explanations of the observed patterns of variation decreases.

Species distributions do not depend on climate only. Geographical range margins might not correspond to the fundamental niche margins [62] and trees might react to non-climate factors such as management history [63], invasive exotic species, or pests [64]. Under these circumstances, the observed growth variations may not represent tree response to climate change and no agreement with predicted responses is expected. In other cases, trees might respond to climate change varying other parameters than those considered. For example, tree height [65] or recruitment rate [66] might be more affected than stem diameter; when this is true, using dendrochronological data might be an ineffective choice. Furthermore, tree ring data might come from unrepresentative specimens, site locations might be unsuitable to catch the global range variation, or species distribution information might be incomplete or at low resolution. Models of habitat suitability might be poor performers, too specific or too sensitive, or thresholds for assigning positive, neutral, and negative responses might be misplaced. In all these cases, the probability of type II errors increases and our approach fails.

As showed, our approach is prone to false negative, failing to reject the null hypothesis even if it is false. In this light, the lack of significant results for *Picea abies* and *Quercus petraea* could mean that one or more of the abovementioned issues prevented us to detect any pattern. It means that the observed responses for *Picea abies* and *Quercus petraea*, which showed a general positive pattern of growth and a stable pattern respectively, could be generated by non-climate linked factors (e.g. different forest management or pest outbreaks) or by their interactions with climate change. It does not necessary imply that these two species are insensitive to climate change, rather it simply shows that our approach failed to disentangle effects from multiple, possibly interacting drivers.

On the other hand, the probability of false positive is very low for our approach, because the level of agreement is due to the joint occurrence of multiple events (site-by-site match of observed and expected trends). As a result, when everything works properly and no factor interferes with the analyses, our spatial approach allows to identify very small differences in geographic patterns of variations. In other words, our results show accurately what is happening in terms of geographic response to climate change by Scots pine and, therefore, which specific climate trajectory is driving such response. Overall, our approach can be interpreted as a photographic film with very low-speed and high-resolution, which works properly only with perfect lighting and setting but, under such conditions, provides extremely detailed images of its subjects.

The capacity of evidencing with high precision the response of natural ecosystems and the relative climatic pressures makes our spatial approach particularly suitable for the implementation of a monitoring/alarm system. This system should consist of a network of sites where dynamic features of populations (e.g. individual growth, demographic trends, reproductive success) are measured for multiple species. The network of sites could be coordinated at a local, regional, or global scale and it would provide detailed background information for adaptation strategies. Indeed, effective strategies for reducing the impacts of climate change (e.g. adaptive management of forests [61], dynamic prioritization of protected areas [67]) require as precise as possible pictures of the stress to be faced. Interestingly, some efforts to set up networks of sites where monitoring and ecological research is carried out on the long run have been made (e.g. the global ILTER network [68] with its regional and national affiliates, the ICPForests monitoring network [69]): these networks could represent a perfect basis for producing the necessary information through the explicit application of our spatial approach. This would only require the collection of some additional dynamic variables and a coordination that explicitly take this approach into account but valuable information for adaptation strategies would be available in return.

Our results show that European ecosystems are facing a very harsh variation of climatic conditions, which is compatible with the top end of all the available GCMs. Instrumental measurements of global CO_2_ concentration already showed that current emission trends continue to track scenarios that lead to the highest temperature increases [70]. Indeed, notwithstanding the international community, in the UNFCCC Conference of Parties (COP21) in Paris, set the target of holding the increase in the global average temperature above pre-industrial levels well below 2°C, with such a level of emissions the rise of global surface temperature for the end of the 21^st^ century is likely to exceed 2°C [4]. In addition, recent analyses suggests that 2015 was 1°C greater than pre-industrial temperatures [71] and that February 2016 exceeded 1.5°C above the base period [72]. Several scientists advise that ‘business as usual’ may produce a 3–5°C increase in global temperatures this century [73,74] and that such level of change can be catastrophic [75]. Global climate is following one specific and extreme trend of variation and natural ecosystems are changing consequently.

In this light, estimating potential impacts of climate change on ecosystems on the basis of the full spectrum of available GCMs, as usually done, can be misleading and unproductive. Indeed, as we showed, future suitability can be over- or under-estimated over a large surface by using the entire set of available GCMs. In addition, natural systems are responding to a climatic variation that is close to the upper limit of the predicted variations. Therefore, considering all the possible scenarios as equally probable, albeit scientifically sound, can drive to underestimate the real level of threat and can push to plan ineffective conservation programs. Instead, as in a case of emergency management, the highest priority should be given to solve the real, specific threat existing. All the other, potential options should be taken for developing secondary plans to put in practice if conditions on the ground change. It means that ecosystem responses should be monitored and the specific process occurring should be identified, allowing to reduce the uncertainty on what is really happening and to improve the applicability of the conservation measures. On our opinion, the time is come for a new generation of ecological studies on climate change impacts, which focuses on a restricted range of future scenarios, representing the real threat the ecosystems are facing in order to support practical and finally effective measures.

## Supporting information

S1 Table Series of ring width data and observed response for *Picea abies*. (DOCX)

S2 Table Series of ring width data and observed response for *Pinus sylvestris*. (DOCX)

S3 Table Series of ring width data and observed response for *Quercus petraea*. (DOCX)

S4 Table Climatic variables retained for ecological niche modelling. (DOCX)

S5 Table Global Climate Models (GCMs) considered. (DOCX)
