# Supplementary material for: Which climate change path are we following? Bad news from Scots pine
Source: PLoS One. 2017 Dec 18;12(12):e0189468. doi: 10.1371/journal.pone.0189468 (PMC5734685; doi:10.1371/journal.pone.0189468)
Supplement: S1 Table — (DOCX) [file pone.0189468.s001.docx]

**S1 Table. Series of ring width data used for *Picea abies*.**

| Location | Code | Most Recent Year | Latitude | Longitude | Observed response |
| --- | --- | --- | --- | --- | --- |
| Matko Kerimäki | finl044 | 2001 | 61.87 | 28.82 | -1 |
| Woody | germ057 | 2001 | 49.2 | 12.38 | 0 |
| Traitsching | germ058 | 2001 | 49.18 | 12.65 | 0 |
| Eichhornberg | germ059 | 2001 | 48.92 | 12.62 | 0 |
| Hessen/Kellerwald dhk35 | germ159 | 2005 | 51.15 | 8.9833 | 1 |
| Pfalz/Westeifel dpe07 | germ162 | 2009 | 50.117 | 6.8667 | 0 |
| Westfalen/Pleiser Hügelland drb38 | germ172 | 2008 | 50.7 | 7.2 | 0 |
| Westfalen/Eifel dre13 | germ185 | 2004 | 50.583 | 6.4667 | 1 |
| Westfalen/Haiger | germ189 | 2005 | 50.717 | 8.1 | 1 |
| Westfalen/Haiger | germ192 | 2005 | 50.85 | 8.2167 | 1 |
| Westfalen/Rothaargebirge dro03 | germ208 | 2005 | 51.183 | 8.4333 | 1 |
| Zagare | lith015 | 2006 | 56.37 | 23.27 | 0 |
| BabiaGora | pola022 | 2004 | 49.583 | 19.05 | 1 |
| DolinaPanszczycka | pola025 | 2004 | 49.25 | 20.0333 | 1 |
| DolinaRygbiego | pola026 | 2004 | 49.233 | 20.0833 | -1 |
| DolinaSuchejwody | pola027 | 2004 | 49.25 | 20.0333 | 1 |
| DolinaWaksmunska | pola028 | 2004 | 49.25 | 20.0667 | 0 |
| Gorce | pola029 | 2003 | 49.55 | 20.1333 | 1 |
| Pilsko | pola030 | 2004 | 49.517 | 19.0333 | 0 |
| RotztokiPodczub | pola032 | 2000 | 49.233 | 20.0833 | -1 |
| RotztokiWooszyn | pola035 | 2000 | 49.233 | 20.0833 | -1 |
| Zolta Turina | pola037 | 2004 | 49.25 | 20.0167 | 1 |
| Ocolasu | roma008 | 2004 | 46.95 | 25.95 | 1 |
| Zanoaga | roma010 | 2004 | 45.35 | 22.7667 | 0 |
| Kosodrovina | svk002 | 2003 | 48.933 | 19.6 | 0 |
| NovaPolinka | svk004 | 2003 | 49.15 | 20.0167 | 0 |
| Tale | svk005 | 2003 | 48.867 | 19.6 | 1 |
| Namdo | swed311 | 2004 | 59.2 | 18.7 | 0 |
| Bjorbo | swed312 | 2005 | 60.45 | 14.73 | 0 |
| Davos GR Sertig | swit179 | 2005 | 46.77 | 9.82 | 1 |
| Bergün GR Val Tuors | swit186 | 2007 | 46.63 | 9.78 | 1 |
| Glarus GL | swit189 | 2007 | 47.03 | 9.07 | 1 |
| Vals GR Riefawald | swit193 | 2008 | 46.62 | 9.2 | 1 |
| Pian di Né Copera | swit226 | 2000 | 46.15 | 8.9931 | 0 |
| Hopflsteig 494 | swit256 | 2005 | 46.3 | 7.7167 | 0 |
| Erlenhoehe | swit258 | 2001 | 47.046 | 8.7133 | 1 |
| Grindelwald BE Mettla | swit279 | 2011 | 46.59 | 7.98 | 1 |
| Saviese | swit329 | 2005 | 46.267 | 7.0333 | 0 |
| San Bernardino | swit332 | 2005 | 46.459 | 9.1833 | 0 |
| Howerla | ukr001 | 2003 | 48.15 | 24.5167 | 0 |
