# Supplementary material for: Which climate change path are we following? Bad news from Scots pine
Source: PLoS One. 2017 Dec 18;12(12):e0189468. doi: 10.1371/journal.pone.0189468 (PMC5734685; doi:10.1371/journal.pone.0189468)
Supplement: S2 Table — (DOCX) [file pone.0189468.s002.docx]

**S2 Table. Series of ring width data used for *Pinus sylvestris*.**

| Location | Code | Most Recent Year | Latitude | Longitude | Observed response |
| --- | --- | --- | --- | --- | --- |
| Karhunpesäkivi Inari | finl021 | 2001 | 68.83 | 27.25 | 1 |
| Kaivanto Paskolahti | finl041 | 2002 | 61.92 | 29 | -1 |
| Punkaharju Esker | finl042 | 2002 | 61.8 | 29.3 | 0 |
| Kaivanto | finl043 | 2001 | 61.93 | 28.98 | -1 |
| Vuotso GSF | finl045 | 2001 | 68.07 | 27.2 | 0 |
| Oulanka National Park Kuusamo | finl046 | 2001 | 66.37 | 29.3 | -1 |
| Kessi Inari | finl047 | 2001 | 68.92 | 28.48 | 0 |
| Keihäsjoki Nature Conservation Area Juuka | finl049 | 2001 | 63.28 | 28.93 | 0 |
| Kukelo Ahmovaara Juuka | finl052 | 2001 | 63.1 | 29.63 | 1 |
| Tutkimusasema | finl054 | 2000 | 67.22 | 26.82 | 0 |
| Vytamoselka | finl055 | 2000 | 67.08 | 27.02 | 0 |
| Ylisjarvi | finl056 | 2000 | 61.93 | 25.72 | 1 |
| Rutajarvi | finl057 | 2000 | 61.92 | 26.02 | 0 |
| Kesänkijärvi | finl069 | 2006 | 67.56 | 24.3 | 0 |
| Luosu PI | finl070 | 2006 | 67.5 | 24.15 | 1 |
| Luosu PS | finl071 | 2006 | 67.5 | 24.15 | 0 |
| Ketomella PK | finl072 | 2006 | 68.22 | 24.05 | 0 |
| Ketomella PT | finl073 | 2006 | 68.22 | 24.05 | 0 |
| Ketomella PW | finl074 | 2006 | 68.22 | 24.05 | 0 |
| Hessen/Kellerwald dhk06 | germ153 | 2005 | 51.15 | 9.0833 | 0 |
| Hessen/Kellerwald dhk36 | germ160 | 2005 | 51.15 | 8.9833 | -1 |
| Westfalen/Eifel dre14 | germ186 | 2005 | 50.583 | 6.4667 | 1 |
| Westfalen/Eifel dre20 | germ187 | 2009 | 50.5 | 6.5667 | 0 |
| Westfalen/Eifel dre29 | germ188 | 2011 | 50.6 | 6.4833 | 0 |
| Westfalen/Siegtal drs01 | germ214 | 2001 | 50.783 | 7.5667 | 0 |
| Westfalen/Ostmünsterland drt03 | germ216 | 2005 | 51.883 | 8.5833 | 1 |
| Spare | latv002 | 2006 | 57.217 | 22.25 | 0 |
| Sirguske | lith016 | 2006 | 53.93 | 23.72 | 0 |
| Karasjok | norw007 | 2001 | 69.42 | 25.63 | -1 |
| Solano de la Vega High | spai060 | 2008 | 40.383 | -0.6833 | 0 |
| Solano de la Vega Low | spai061 | 2008 | 40.383 | -0.7 | 0 |
| Puerto de Gudar Low | spai062 | 2008 | 40.35 | -0.6833 | 0 |
| Puerto de Gudar High | spai063 | 2008 | 40.35 | -0.7 | 0 |
| Pamplona | spai070 | 2005 | 42.1 | -1.1333 | 0 |
| Cercedilla M rid | spai071 | 2008 | 40.785 | -4.0392 | 0 |
| Cercedilla M rid VA | spai072 | 2008 | 40.788 | -4.0411 | -1 |
| Popr | svk006 | 2006 | 48.917 | 20.2833 | 0 |
| Bjorbo | swed305 | 2002 | 60.45 | 14.73 | -1 |
| Lerbäck, Askersund | swed307 | 2004 | 58.95 | 15.03 | 0 |
| Saltsjob en | swed308 | 2003 | 59.28 | 18.3 | 0 |
| Aneby, Smaland Bredesta/Lysing | swed309 | 2003 | 57.9 | 14.9 | 0 |
| Rälla Tall, Högsrum sn, Öland | swed313 | 2006 | 56.77 | 16.55 | -1 |
| Nonshaugen | swed327 | 2000 | 64.45 | 13.97 | 0 |
| Tyresta | swed328 | 2000 | 59.18 | 18.27 | 1 |
| Kiruna | swed330 | 2006 | 67.9 | 20.1 | 0 |
| Kiruna KW | swed331 | 2006 | 67.9 | 20.1 | 0 |
| Torneträsk TR | swed332 | 2005 | 68.2 | 19.8 | 0 |
| Torneträsk TW | swed333 | 2006 | 68.2 | 19.8 | 0 |
| Sierre VS Pfynwald | swit188 | 2007 | 46.3 | 7.57 | 0 |
| Scheni Biela | swit209 | 2005 | 46.383 | 8.1 | 1 |
| Rusc a Copera | swit228 | 2000 | 46.15 | 8.9931 | 0 |
| Deischbach | swit231 | 2005 | 46.383 | 8.1 | 1 |
| Felsberg GR Brennten | swit269 | 2009 | 46.85 | 9.4667 | 0 |
| Riddes | swit321 | 2005 | 46.15 | 7.2167 | -1 |
| Ritena | swit328 | 2005 | 46.383 | 8.1 | -1 |
| Saviese | swit330 | 2005 | 46.267 | 7.0333 | 0 |
| Vigera | swit348 | 2002 | 46.5 | 8.7667 | 1 |
