# Supplementary material for: Which climate change path are we following? Bad news from Scots pine
Source: PLoS One. 2017 Dec 18;12(12):e0189468. doi: 10.1371/journal.pone.0189468 (PMC5734685; doi:10.1371/journal.pone.0189468)
Supplement: S3 Table — (DOCX) [file pone.0189468.s003.docx]

**S3 Table. Series of ring width data used for *Quercus petraea*.**

| Location | Code | Most Recent Year | Latitude | Longitude | Observed response |
| --- | --- | --- | --- | --- | --- |
| Hessen/Kellerwald dhk03 | germ150 | 2005 | 51.167 | 8.9667 | 0 |
| Hessen/Kellerwald dhk08 | germ154 | 2005 | 51.15 | 9.0833 | 0 |
| Hessen/Kellerwald dhk09 | germ155 | 2005 | 51.15 | 9.0833 | -1 |
| Hessen/Kellerwald dhk10 | germ156 | 2005 | 51.15 | 9.0833 | 0 |
| Hessen/Kellerwald dhk11 | germ157 | 2005 | 51.15 | 9.0667 | 0 |
| Hessen/Kellerwald dhk12 | germ158 | 2005 | 51.15 | 9.0666 | 0 |
| Westfalen/Siebengebirge drb06 | germ165 | 2004 | 50.667 | 7.2333 | 1 |
| Westfalen/Siebengebirge drb07 | germ166 | 2004 | 50.667 | 7.2333 | 0 |
| Westfalen/Siebengebirge drb34 | germ171 | 2005 | 50.683 | 7.2167 | -1 |
| Westfalen/Eifel dre01 | germ173 | 2004 | 50.65 | 6.3167 | 1 |
| Westfalen/Eifel dre06 | germ178 | 2004 | 50.433 | 6.55 | 0 |
| Westfalen/Eifel dre07 | germ179 | 2004 | 50.667 | 6.2667 | 0 |
| Westfalen/Eifel dre09 | germ181 | 2004 | 50.6 | 6.4167 | 0 |
| Westfalen/Eifel dre11 | germ183 | 2004 | 50.567 | 6.35 | -1 |
| Westfalen/Haiger | germ191 | 2005 | 50.717 | 8.1167 | 0 |
| Westfalen/Haiger | germ194 | 2005 | 50.85 | 8.2167 | 0 |
| Westfalen/Köln | germ197 | 2005 | 51.033 | 6.8 | 1 |
| Westfalen/Oberbergisches drl04 | germ202 | 2005 | 50.967 | 7.7167 | 0 |
| Niederrheinisches Tiefland drn20 | germ205 | 2009 | 51.75 | 6.05 | -1 |
| Westfalen/Arnsberg dro02 | germ207 | 2005 | 51.1 | 8.0167 | -1 |
| Cavergno | swit230 | 2002 | 46.35 | 8.6 | 0 |
| Ruschein GR La Caglia | swit270 | 2009 | 46.783 | 9.1833 | 0 |
| Tamins GR Eichwald | swit271 | 2009 | 46.833 | 9.4333 | 0 |
| Sihlwald | swit335 | 2006 | 47.269 | 8.5608 | 0 |
| Cugnasco | swit346 | 2006 | 46.185 | 8.8817 | 0 |
| Urdorf | swit364 | 2006 | 46.322 | 7.5619 | 0 |
