# Supplementary material for: Which climate change path are we following? Bad news from Scots pine
Source: PLoS One. 2017 Dec 18;12(12):e0189468. doi: 10.1371/journal.pone.0189468 (PMC5734685; doi:10.1371/journal.pone.0189468)
Supplement: S4 Table — (DOCX) [file pone.0189468.s004.docx]

**S4 Table. Climatic variables retained for ecological niche modeling.**

| Variable code and name |
| --- |
| BIO1 = Annual Mean Temperature |
| BIO2 = Mean Diurnal Range (Mean of monthly (max temp - min temp)) |
| BIO4 = Temperature Seasonality (standard deviation *100) |
| BIO8 = Mean Temperature of Wettest Quarter |
| BIO9 = Mean Temperature of Driest Quarter |
| BIO15 = Precipitation Seasonality (Coefficient of Variation) |
| BIO18 = Precipitation of Warmest Quarter |
| BIO19 = Precipitation of Coldest Quarter |
