# Supplementary material for: Which climate change path are we following? Bad news from Scots pine
Source: PLoS One. 2017 Dec 18;12(12):e0189468. doi: 10.1371/journal.pone.0189468 (PMC5734685; doi:10.1371/journal.pone.0189468)
Supplement: S5 Table — (DOCX) [file pone.0189468.s005.docx]

**S5 Table. Global Climate Models (GCMs) considered.**

| GCM | code | Institution |
| --- | --- | --- |
| ACCESS1-0 | AC | CSIRO (Commonwealth Scientific and Industrial Research Organisation, Australia), and BOM (Bureau of Meteorology, Australia) |
| BCC-CSM1-1 | BC | Beijing Climate Center, China Meteorological Administration |
| CCSM4 | CC | National Center for Atmospheric Research |
| CESM1-CAM5-1-FV2 | CE | National Science Foundation, Department of Energy, National Center for Atmospheric Research |
| CNRM-CM5 | CN | Centre National de Recherches Meteorologiques / Centre Europeen de Recherche et Formation Avancees en Calcul Scientifique |
| GFDL-CM3 | GF | Geophysical Fluid Dynamics Laboratory |
| GFDL-ESM2G | GD | Geophysical Fluid Dynamics Laboratory |
| GISS-E2-R | GS | NASA Goddard Institute for Space Studies |
| HadGEM2-AO | HD | National Institute of Meteorological Research/Korea Meteorological Administration |
| HadGEM2-CC | HG | Met Office Hadley Centre (additional HadGEM2-ES realizations contributed by Instituto Nacional de Pesquisas Espaciais) |
| HadGEM2-ES | HE | Met Office Hadley Centre (additional HadGEM2-ES realizations contributed by Instituto Nacional de Pesquisas Espaciais) |
| INMCM4 | IN | Institute for Numerical Mathematics |
| IPSL-CM5A-LR | IP | Institut Pierre-Simon Laplace |
| MIROC-ESM-CHEM | MI | Japan Agency for Marine-Earth Science and Technology, Atmosphere and Ocean Research Institute (The University of Tokyo), and National Institute for Environmental Studies |
| MIROC-ESM | MR | Japan Agency for Marine-Earth Science and Technology, Atmosphere and Ocean Research Institute (The University of Tokyo), and National Institute for Environmental Studies |
| MIROC5 | MC | Atmosphere and Ocean Research Institute (The University of Tokyo), National Institute for Environmental Studies, and Japan Agency for Marine-Earth Science and Technology |
| MPI-ESM-LR | MP | Max Planck Institute for Meteorology (MPI-M) |
| MRI-CGCM3 | MG | Meteorological Research Institute |
| NorESM1-M | NO | Norwegian Climate Centre |
